# Association between education and future leisure-time physical inactivity: a study of Finnish twins over a 35-year follow-up

**DOI:** 10.1186/s12889-016-3410-5

**Published:** 2016-08-04

**Authors:** Maarit Piirtola, Jaakko Kaprio, Urho M. Kujala, Kauko Heikkilä, Markku Koskenvuo, Pia Svedberg, Karri Silventoinen, Annina Ropponen

**Affiliations:** 1 Department of Public Health, University of Helsinki, PO Box 41 (Tukholmankatu 8, 2B), FI-00014 Helsinki, Finland; 2 Institute for Molecular Medicine Finland (FIMM), University of Helsinki, Helsinki, Finland; 3 Department of Health, National Institute for Health and Welfare, Helsinki, Finland; 4 Department of Health Sciences, University of Jyväskylä, Jyväskylä, Finland; 5 Department of Clinical Neuroscience, Division of Insurance Medicine, Karolinska Institutet, Stockholm, Sweden; 6 Department of Social Research, Population Research Unit, University of Helsinki, Helsinki, Finland; 7 Finnish Institute of Occupational Health, Helsinki, Finland

**Keywords:** Adult, Behavioral genetics, Cohort studies, Educational status, Exercise, Follow-Up studies, Twins

## Abstract

**Background:**

Education is associated with health related lifestyle choices including leisure-time physical inactivity. However, the longitudinal associations between education and inactivity merit further studies. We investigated the association between education and leisure-time physical inactivity over a 35-year follow-up with four time points controlling for multiple covariates including familial confounding.

**Methods:**

This study of the population-based Finnish Twin Cohort consisted of 5254 twin individuals born in 1945–1957 (59 % women), of which 1604 were complete same-sexed twin pairs. Data on leisure-time physical activity and multiple covariates was available from four surveys conducted in 1975, 1981, 1990 and 2011 (response rates 72 to 89 %). The association between years of education and leisure-time physical inactivity (<1.5 metabolic equivalent hours/day) was first analysed for each survey. Then, the role of education was investigated for 15-year and 35-year inactivity periods in the longitudinal analyses. The co-twin control design was used to analyse the potential familial confounding of the effects. All analyses were conducted with and without multiple covariates. Odds Ratios (OR) with 95 % Confidence Intervals (CI) were calculated using logistic and conditional (fixed-effects) regression models.

**Results:**

Each additional year of education was associated with less inactivity (OR 0.94 to 0.95, 95 % CI 0.92, 0.99) in the cross-sectional age- and sex-adjusted analyses. The associations of education with inactivity in the 15- and 35-year follow-ups showed a similar trend: OR 0.97 (95 % CI 0.93, 1.00) and OR 0.94 (95 % CI 0.91, 0.98), respectively. In all co-twin control analyses, each year of higher education was associated with a reduced likelihood of inactivity suggesting direct effect (i.e. independent from familial confounding) of education on inactivity. However, the point estimates were lower than in the individual-level analyses. Adjustment for multiple covariates did not change these associations.

**Conclusions:**

Higher education is associated with lower odds of leisure-time physical inactivity during the three-decade follow-up. The association was found after adjusting for several confounders, including familial factors. Hence, the results point to the conclusion that education has an independent role in the development of long-term physical inactivity and tailored efforts to promote physical activity among lower educated people would be needed throughout adulthood.

## Background

Physical inactivity is related to a variety of chronic conditions, such as cardiovascular diseases, type 2 diabetes and some cancers [1–3], resulting in increased health care costs [4, 5] and the risk of premature death [1, 3, 6, 7]. Therefore, physical inactivity is regarded as a major global public health problem [1–3]. Globally, the prevalence of physical inactivity (i.e. those who do not meet physical activity guidelines [8, 9]) has varied from 17 to 31 % in adult populations [6, 10]. Even though several domains of physical activity (e.g. leisure-time, commuting, occupational and domestic activities) have been shown to have beneficial associations for health [9] and reduced all-cause mortality [11], one of the strongest and most reported associations has been with leisure-time physical activity (LTPA) [11, 12].

Education has shown to be strongly associated with health-related behaviours, including time spent in LTPA [13, 14]. Compared to those with lower education, highly educated individuals are more likely to have better adult health and a healthier lifestyle [13, 15, 16], including more LTPA [17–20]. Furthermore, the association between high formal education and higher level of LTPA in adults has been confirmed in longitudinal studies [17, 19, 21, 22]. A study with a 10-year follow-up among Dutch adults indicated that higher education was related to remaining active compared to becoming inactive [22], and another Dutch study with a 6-year follow-up among the 15–74-year old population showed that those with a lower education were more likely to decrease their level of LTPA than those with a higher education [21]. Furthermore, in a short (2-year) follow-up, an increase in education increased physical activity among US working age population [17]. In addition, in a 16-year follow-up higher education prevented a decrease in LTPA among the Canadian adult population [19]. The positive effect of education on a higher level of LTPA has also been consistent in cross-sectional studies [16, 18, 20, 23, 24]. However, in order to improve timing and focus of promoting LTPA during the life span, the role of education in long-term physical inactivity or in the change from being active to inactive merits further studies.

An aspect that may play an important role in the association between education and LTPA is the stability of health behaviours. LTPA has been reported to remain moderately stable across the lifespan [25, 26]. Being active in one’s leisure time already in young adulthood seems to increase the likelihood of being physically active also later in life [26]. However, many people do change their LTPA behaviour during adulthood [19, 22, 27, 28]. In a Finnish cohort study following 5254 participants from early adulthood to retirement age and utilizing four surveys, the majority of people changed their LTPA behaviour during the 35-year follow-up [28]. The prevalence of inactivity showed a decreasing trend from 56 % in 1975 to 36 % in 2011, and only 9 % of the participants were persistently inactive in all four surveys [28]. In order to capture the trend of long-term LTPA behaviour and to plan actions to promote life-long physical activity, further follow-up studies are needed about the effect of education on LTPA behaviour formation that employ a follow-up over decades with several data collection points.

Along with a longitudinal association between education and LTPA, the role of familial confounding between these factors is important. Both educational level and physical activity aggregate in families, with some evidence of genetics on both factors [29–31]. Twin studies are valuable in assessing the role of familial (genetic factors and shared environmental experiences and exposures, particularly in childhood) and unique environmental factors in associations between factors of interest [32]. Twin pairs raised together, such as common siblings, have the same family background, and thus they share many environmental exposures in childhood, such as the parents’ socioeconomic status. Dizygotic (DZ) twin pairs also share on average 50 % of their segregating genes, whereas monozygotic (MZ) twin pairs are virtually identical on the gene sequence level. The co-twin control design of twin pairs discordant for a factor of interest [32, 33] provides an opportunity to control for familial effects on the associations between different factors such as education and physical inactivity in this study. Hence, twin pairs discordant for physical inactivity would provide the possibility to determine whether the association between education and inactivity is influenced by familial confounding. If familial factors play a role, the association between education and inactivity should exist in the analyses of the whole cohort (i.e. twins treated as individuals) but not between discordant co-twins. This is why discordant analyses should always be interpreted by comparing them with the results of all individuals. In contrast, if genetics plays a role, then the association should be present within DZ twin pairs, but not within MZ twin pairs. Further, if the association is found both within MZ and DZ pairs, the finding would suggest independence from familial factors and reflect a somewhat direct association between education and physical inactivity [33–35].

A weak protective association of higher education with physical inactivity has been found in cross-sectional twin studies controlling for familial confounding, specifically, the genetic and environmental factors shared by co-twins [15, 36–38]. Longitudinal twin studies investigating the association between education and leisure-time physical inactivity are rare, but this type of study can shed light into the nature of the association between education and LTPA. Hence, our hypothesis is that those with a higher education would be less likely to remain inactive during the 35-year follow-up; we expect to see this association also within discordant twin pairs, controlling for familial confounding.

## Methods

The aim of this study was to investigate the association between education and leisure-time physical inactivity among Finnish adult twins with follow-up data from 1975 to 2011 as well as the influence of familial factors on this association.

### Sample

The data were derived from the Older Finnish Twin Cohort of same-sex twins born before 1958 with four postal surveys conducted in 1975, 1981, 1990 and 2011 [39, 40]. The participation rates in the surveys varied between 72 and 89 % [28]. The Older Finnish Twin Cohort data collection has been described in detail earlier [28]. For this study, only individuals born between 1945 and 1957 were included because the fourth survey only targeted this age group. In total, 5575 participants answered all four surveys. Among the respondents, 321 individuals (6 % of the total sample) had missing or incomplete LTPA data for MET calculations in at least one time point and were therefore not included in the final sample. In 1975, the mean age of the 321 (51 % men) excluded individuals was 24.6 years (SD 0.05), and of those included in the analyses 23.9 (SD 0.21), (*p* = 0.001). Those who were not included had on average 1.18 (SD 0.15) years less education (*p* < 0.001) and had a 0.32 (SD 0.16) higher BMI (*p* = 0.042), but no differences were seen in reported leisure-time physical inactivity (*p* = 0.127), smoking status (*p* = 0.236) or mean alcohol consumption (*p* = 0.459) compared to the 5254 included participants in 1975. The data of 5254 individuals (41 % men) included 1604 complete twin pairs: 588 MZ, 944 DZ and 72 pairs of unknown zygosity.

**Fig. 1:**
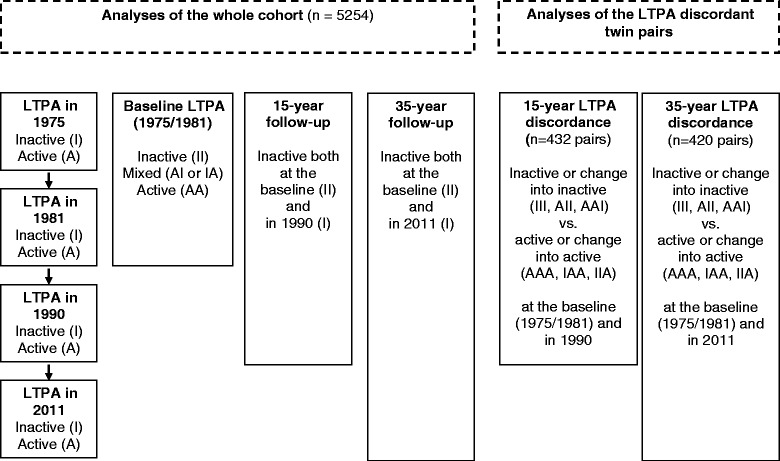
Categorization of leisure-time physical activity (LTPA) at baseline (1975/1981), in 1990 and in 2011, and in analysing long-term inactivity both in the whole cohort (*n* = 5254) and in the long-term LTPA discordant twin pairs. A = active (> 1.5 MET h/day), I = inactive (≤ 1.5 MET h/day)

*Leisure-time physical inactivity* was defined under a globally recommended level of physical activity [3, 8, 9]. The questions about LTPA and the calculation for the MET index for this cohort have been described in detail earlier [28, 41]. Briefly, leisure-time physical exercise was queried in all four surveys and consisted of responses on the frequency (per month), duration (per session) and intensity of exercise by asking, “Is your leisure-time physical exercise on average as intensive as…”, with four response alternatives: walking (4 METs), walking and jogging (6 MET), jogging (10 METs) or running (13 METs). Surveys conducted in 1975, 1980 and 2011 also included a specific question about the average exercise per year. In order to minimize social desirability [42] and the impact of seasonal variation [43] on exercising, participants who reported “practically no exercise at all” per a year were categorized as having zero METs for their exercise MET per month, regardless of their answers to duration, intensity and frequency of exercise questions. A separate question gathered the daily time of commuting by physically active means (walking, jogging and cycling) to and from work (4 METs), except in 1990 when exercise and commuting were combined [28]. The MET indexes for exercise and commuting activities were calculated by multiplying the general intensity and the average duration and frequency of activities at each time point separately [7, 28, 41, 44], transformed into average MET hours per day and summed together to receive the total LTPA METs. In a sub-study conducted in 2005, the questionnaire-based exercise MET index of this study (identical with the 1975 and 1981 questionnaires) has shown moderate agreement (the intraclass correlation (ICC) of 0.68) with the exercise MET index based on a comprehensive structured face-to-face interview on all possible specific modes of LTPA and their structured frequency, duration and intensity descriptions, excluding commuting [45]. For commuting activities, the ICC was 0.93. For this study, leisure-time physical inactivity (I) at each time point was defined as the average daily energy expenditure being less than 1.5 MET hours (no more than 10.5 MET hours/week), utilizing the cut-off point of 10 MET hours/week for inactive persons [1, 3, 8]. Others were defined as physically active (A). In order to follow those who remain or become inactive during the years, the baseline LTPA status was formed using the data from the first two surveys: those who were consistently inactive in both the 1975 and 1981 surveys (inactive [II]), those who had changed their activity status (from active to inactive [AI] or from inactive to active [IA]) and those who were active in both surveys (active [AA]) (Fig. 1).

Completed *formal education* was queried in 1975 and 1981 by one question: “What kind of education have you had, and what courses have you attended?” The respondents were asked to classify themselves into one of eight educational categories, which were converted into years of education: less than primary school (3 years of education), primary school (6 years), primary school and at least one year of education such as vocational training (7 years), secondary school (9 years), secondary school and at least one year of education (10 years), high school graduate (12 years), high school and at least one year of education (13 years), and a university degree (16 years) [46]. We used the latest enquiry of education (1981) as the main exposure variable since at that time the youngest participants were 24 years of age and most of the participants had achieved the majority of their formal education.

All covariates, except age at each survey, were included from the 1975 survey [47]. *Socioeconomic status* was based on self-reported main lifetime occupation in 1975, classified by the 1970 Finnish census classification of occupational and social class with six main categories: upper and lower white-collar workers, skilled and unskilled manual workers, farmers, and others (including students, conscripts, full-time homemakers and those otherwise not classified). Those not employed at the baseline were asked to report their previous occupation. *Marital status* was reclassified into two categories: single, divorced or widowed, and married or co-habiting. A dichotomous variable of *working status* (at work, not at work) was determined from two questions of overall working status (working, retired, retired on disability pension, unemployed or other) and work status at the moment of the survey (employee, entrepreneur, farmer, currently unemployed, never employed). *Body mass index (BMI)* was computed from self-reported weight and height (kg/m^2^). The validity of BMI values has been shown to be high in this cohort [48]. *Alcohol consumption* was measured with several questions on the quantity of beer, wine and spirits used in an average week or month and converted into grams of absolute alcohol per day. *Cigarette smoking* was queried in detail with a series of questions and categorized as has been done previously, including answers from all the surveys, as never smoker, former smoker and current smoker.

### Analyses

The main focus of the analyses was the association of average years of education in 1981 with leisure-time physical inactivity both in 1975 and 1981 (baseline) and with the continuing status of inactive in 1990 or 2011 (long-term inactivity). We also tested the association of education with a change to inactivity over the years in those determined as active at the baseline and but having an inactive status either in 1990 or 2011. However, the associations for a change to inactivity were similar to the associations for long-term inactivity. Hence, only the results for long-term inactivity will be reported.

First, we analysed the cross-sectional association between education in 1981 and inactivity at baseline (1975/1981). Then, we conducted longitudinal analyses of the associations between education and inactivity at baseline and continued inactivity in 1990 (15-year inactivity period) or 2011 (35-year inactivity period) (Fig. 1). The analyses for 2011 were performed both taking into account the LTPA level in 1990 and without that level. Logistic regression models with Odds Ratios (OR) and 95 % Confidence Intervals (95 % CI) were used for the analyses of the whole cohort when twins were treated as individuals. In all individual-level analyses, the effect of the clustered sample design, in other words the use of twin pairs rather than unrelated individuals, on the standard error was taken into account using the cluster option to obtain robust standard errors. Socioeconomic status was tested as a covariate but was excluded from the final multivariate analyses because it did not have any effect on the associations between education and inactivity.

The co-twin control design was used to consider potential familial confounding on the effect of education on physical inactivity in the analyses [33]. First, we analysed the association between education and inactivity in twin pairs discordant for LTPA (active vs. inactive) at each survey for 1981, 1990 and 2011. Then, we performed longitudinal within-pair analyses separately among twin pairs discordant for LTPA over a 15-year (from 1975/1981 to 1990) and a 35-year (from 1975/1981 to 2011) follow-up. For the longitudinal analyses, the long-term LTPA behaviour was dichotomized into broadly inactive (persistently inactive, mainly inactive and a change from active to inactive) and broadly active (persistently active, mainly active and a change from inactive to active) to maintain a sufficient amount of discordant pairs and statistical power in the analyses (Fig. 1). Those with mixed LTPA behaviour (IAI or AIA) during the follow-ups were excluded from these discordant pair analyses (*n* = 841 in 1990, *n* = 861 in 2011). The within-pair analyses were conducted for all discordant twin pairs first and then separately for MZ and DZ pairs. Finally, all the within-pair analyses were conducted with multiple covariates. The within-pair analyses were performed using conditional (fixed-effects) logistic regression.

In the analyses for each survey (1981, 1990 and 2011), we identified 632 to 671 twin pairs discordant for LTPA, including 33 and 34 pairs with uncertain zygosity. The number of discordant twin pairs was between 401 and 424 in DZ pairs and between 198 and 226 in MZ pairs. The numbers of LTPA discordant pairs in the longitudinal analyses were 432 (263 DZ and 151 MZ pairs) for the 15-year discordance (from 1975 to 1990) and 420 (266 DZ and 132 MZ pairs) for the 35-year discordance (from 1975 to 2011).

Stata SE version 13.1 (StataCorp, College Station, Texas, USA) was used for the analyses. In all analyses, the active group was used as a reference group.

### Sensitivity analyses

In the sensitivity analyses, we tested the effect of being on a disability pension (*n* = 26) in 1975 and the working status from 1975 to 2011 (ability to work at all four time points) on the results as indicators of health. The working status from 1975 to 2011, covering 35 years of being healthy enough to work, included either being at work, studying or seeking a job for all surveys. The analyses were done separately with and without old age pensioners in 2011 (*n* = 1243). The sensitivity analyses did not reveal any effects on the results. Hence, the results will be reported for all individuals.

## Results

The mean age of the 5254 participants (59 % women) was 23.9 years (range 18 to 31 years) in 1975 and 60.3 years (range 53 to 67 years) in 2011. The mean years of education in 1981 was 8.9 (SD 3.1) in men and 9.0 (SD 3.0) in women. In both sexes, the median number of education years in 1981 was seven (range 3 to 16 years). At baseline (1975/1981), 33 % of the participants (31 % of men and 34 % of women) were persistently inactive (Table 1).

**Table 1:** Characteristics of the background factors by leisure-time physical activity (LTPA) status in 1975 and in 1981

Background factors	Persistently active in 1975 and in 1981 (AA; *n* = 1545)	Change in LTPA status between 1975 and 1981 (IA or AI, *n* = 1981)	Persistently inactive in 1975 and in 1981 (II, *n* = 1728)
	mean (SD)	mean (SD)	mean (SD)
Age in 1975 (years)	24.1 (3.7)	23.7 (3.8)	23.8 (3.8)
Body Mass Index in 1975 (kg/m^2)	21.5 (2.5)	21.5 (2.7)	21.7 (2.9)
Alcohol consumption in 1975 (g /day)	7.2 (9.6)	7.0 (10.2)	7.0 (10.1)
Education years in 1981, mean (SD), median (range)	9.2 (3.2), 7 (3-16)	9.0 (3.1), 7 (6-16)	8.6 (2.9), 7 (3-16)
Education level in 1981	%	%	%
Less than primary school (3 years)	0	0	0
Primary school (6 years)	19	22	26
Primary school + at least 1 year vocational school (7 years)	31	31	31
Secondary school (9 years)	8	9	8
Secondary school + at least 1 year vocational school (10 years)	17	16	16
Upper secondary school (12 years)	2	2	2
Upper secondary school + at least 1 year vocational school (13 years)	12	13	12
University degree (16 years)	10	8	5
Social class in 1975			
Upper white collar	7	5	4
Lower white collar	32	27	25
Skilled worker	36	36	40
Unskilled worker	8	10	9
Farmer	3	3	5
Other	14	18	17
Marital Status in 1975			
Married or co-habiting	41	41	46
Single, divorced or widowed	59	59	54
Working status in 1975			
At work	77	65	59
Not at work	23	35	41
Smoking status in 1975			
Never smoker	53	49	45
Former smoker	17	15	15
Current smoker	30	36	40

Persistently active (LTPA energy cost > 1.5 MET hours per day both in 1975 and in 1981); persistently inactive (LTPA energy cost ≤ 1.5 MET hours per day both in 1975 and in 1981). A = active, I = inactive

The proportion of inactive individuals decreased from 47 % in 1981 to 34 % in 1990 and to 36 % in 2011 (Table 2). In all time points, the mean education years were 0.5 to 0.6 years lower in physically inactive than in active individuals.

**Table 2:** Cross-sectional analyses of inactivity and education at each survey. Proportions of inactivity and means of education years in 1981 by leisure-time physical activity (LTPA) and the associations of each year of additional education (Odds Ratios [OR] and 95 % Confidence Intervals [95 % CI]) with being physically inactive at the survey in all participants and in twin pairs discordant for LTPA

LTPA status at survey	% (n)	Education years in 1981	All participants (*n* = 5254)*		Discordant twin pairs ** ^a^		
			Age- and sex-adjusted model	Multivariate model ^b^	All pairs	DZ pairs	MZ pairs
		Mean (95 % CI)	OR (95 % CI)	OR (95 % CI)	OR (95 % CI)	OR (95 % CI)	OR (95 % CI)
1981							
Active	53 (2762)	9.19 (9.08, 9.32)	1.00 (reference)	1.00	1.00	1.00	1.00
Inactive	47 (2492)	8.68 (8.57, 8.79)	0.95 (0.93, 0.96)	0.95 (0.93, 0.97) ^c^	0.94 (0.88, 1.00)	0.93 (0.87, 1.00)	0.94 (0.80, 1.09)
1990							
Active	66 (3446)	9.13 (9.03, 9.23)	1.00	1.00	1.00	1.00	1.00
Inactive	34 (1808)	8.61 (8.48, 8.74)	0.95 (0.93, 0.97)	0.96 (0.94, 0.99) ^d^	0.92 (0.87, 0.98)	0.93 (0.87, 1.00)	0.85 (0.73, 0.99)
2011							
Active	64 (3339)	9.18 (9.08, 9.30)	1.00	1.00	1.00	1.00	1.00
Inactive	36 (1915)	8.55 (8.41, 8.67)	0.94 (0.92, 0.96)	0.97 (0.95, 0.99) ^e^	0.94 (0.89, 1.00)	0.95 (0.89, 1.02)	0.86 (0.72, 1.03)

LTPA: leisure-time physical activity; inactive: LTPA energy cost ≤1.5 MET h/day; active (reference group): LTPA energy cost >1.5 MET h/day

*Logistic regression model, ** Conditional logistic regression model

a) Twin pairs discordant for LTPA (active vs. inactive) at each survey. Numbers of discordant pairs in the basic models (age and sex automatically adjusted in the same-sexed twin pairs) were 671 in 1981 (424 DZ and 213 MZ pairs), 664 in 1990 (404 DZ and 226 MZ pairs) and 632 in 2011 (401 DZ and 198 MZ pairs)

b) Sex, age at the survey, Body Mass Index in 1975, working status in 1975, marital status in 1975, alcohol use (g /day) in 1975 and smoking status in 1975 were included as covariates

c) In addition to other covariates, LTPA status in 1975 has been included as a covariate

d) In addition to other covariates, LTPA status from 1975 to 1981 has been included as a covariate

e) In addition to other covariates, LTPA status from 1975 to 1981 and LTPA status in 1990 has been included as covariates

Cross-sectional analyses showed that each additional year of education was associated with lower odds of being inactive in 1981 in the age- and sex-adjusted models (OR 0.95; 95 % CI, 0.93, 0.96). The ORs for inactivity were a similar magnitude in 1990 and in 2011 (Table 2). In the multivariate analyses, the association remained, and each additional year of education lowered the likelihood for being physically inactive (OR 0.95 in 1981, OR 0.96 in 1990, and OR 0.97 in 2011).

In the corresponding cross-sectional within-pair analyses of all twin pairs discordant for their LTPA in 1981, each additional year of education was still associated with lower odds of being inactive (OR 0.94; 95 % CI, 0.88, 1.00) compared to the active co-twin (Table 2). The ORs for all twin pairs were 0.92 in 1990 (95 % CI, 0.87, 0.98) and 0.94 (95 % CI, 0.89, 1.00) in 2011. The ORs were at the same magnitude and direction in MZ and in DZ twin pairs in all time points although with wider CIs. Adjustment for multiple covariates confirmed these associations (data not shown).

Longitudinal analyses among all individuals showed that among those who were inactive in both 1975 and 1981, higher education was associated with a lower likelihood for being inactive also in 1990, representing a 15-year inactivity period (OR 0.97; 95 % CI, 0.93, 1.00) (Table 3). The OR for 35 years of inactivity was 0.94 with or without adjustment for LTPA status in 1990. Multivariate analyses confirmed these associations.

**Table 3:** Association of each year of additional education (Odds Ratios [OR] and 95 % Confidence Intervals [95 % CI]) with a 15-year physical inactivity period (in 1990) and a 35-year inactivity period (in 2011) among participants already physically inactive at baseline (1975/1981) and among twin pairs with long-term inactive behaviour

Long-term physical inactivity	All participants (*n* = 5254) *		Discordant twin pairs ** ^a^					
	Age- and sex-adjusted model	Multivariate model ^b^	Basic model ^b^			Multivariate model ^b, c^		
			All pairs	DZ pairs	MZ pairs	All pairs	DZ pairs	MZ pairs
	OR (95 % CI)	OR (95 % CI)	OR (95 % CI)	OR (95 % CI)	OR (95 % CI)	OR (95 % CI)	OR (95 % CI)	OR (95 % CI)
Inactive in 1990	0.97 (0.93, 1.00)	0.97 (0.93, 1.00)	0.93 (0.87, 1.00)	0.94 (0.86, 1.02)	0.83 (0.70, 1.00)	0.93 (0.86, 1.01)	0.98 (0.89, 1.07)	0.69 (0.54, 0.87)
Inactive in 2011	0.94 (0.91, 0.97)	0.95 (0.91, 0.98)	0.96 (0.89, 1.03)	0.97 (0.89, 1.06)	0.83 (0.66, 1.04)	0.98 (0.91, 1.06)	1.01 (0.92, 1.10)	0.84 (0.67, 1.05)
Inactive in 2011 ^d^	0.94 (0.91, 0.98)	0.95 (0.92, 0.99)	0.96 (0.90, 1.05)	0.99 (0.91, 1.07)	0.85 (0.68, 1.06)	0.95 (0.45, 1.98)	1.01 (0.93, 1.10)	0.87 (0.69, 1.09)

In each survey (1975, 1981, 1990, 2011), inactive: LTPA energy cost ≤1.5 MET h/day; active: LTPA energy cost >1.5 MET h/day. The reference group in the individual-based analyses is those of active in 1990 or in 2011

*Logistic regression model, ** Conditional logistic regression model

a) Co-twin control analyses for twin pairs long-term discordant for LTPA, i.e. broadly inactive (persistently inactive, mainly inactive and a change from active to inactive) vs. broadly active (persistently active, mainly active and a change from inactive to active) in 1975–1981/1990 or in 1975–1981/2011. The numbers of long-term LTPA discordant pairs in the basic models were 432 in 1975–1981/1990 (263 DZ and 151 MZ pairs) and 420 in 1975–1981/2011 (266 DZ and 132 MZ pairs). The reference group in the co-twin control analyses is broadly active

b) Age and sex are automatically adjusted in the same-sexed twin pairs

c) Body Mass Index in 1975, working status in 1975, marital status in 1975, alcohol use (g/day) in 1975 and smoking status in 1975 were included as covariates

d) LTPA in 1990 included as a covariate

In the longitudinal within-pair analyses, the ORs for each year of higher education were 0.93 (95 % CI, 0.87, 1.00) for the 15-year inactivity period and 0.96 (95 % CI, 0.87, 1.03) for the 35-year inactivity period (Table 3). In LTPA discordant MZ and DZ twin pairs, the ORs were at the same magnitude. Adjustment for multiple covariates did not change the associations.

## Discussion

This cohort study of 5254 twin individuals over a 35-year follow-up with four comprehensive surveys contributes to the small body of longitudinal twin studies about the association between formal education and long-term leisure-time physical inactivity. In this study, we were also able to address possible familial confounding by using a co-twin design. Our results indicated that each additional year of education was associated with a lesser likelihood of being physically inactive at each time point, and the association remained in the prospective 15- and 35-year follow-ups. Adjustment for multiple covariates had only a minor effect on the associations, indicating the independent role of education in the formation of LTPA. Our findings support findings from the earlier cross-sectional studies [16, 18, 20, 23, 24] that have shown leisure-time physical inactivity to be more common among those with lower educational attainment. In particular, our results shed more light into the previous longitudinal studies that did not adjust for familial factors, in which higher education has been associated with a long-term higher level or the positive development of LTPA [17, 19, 21, 22]. However, LTPA has comprised different domains of activities (light physical activity, exercise, and sports, with and without commuting etc.) in different studies [2, 18]. Further, there is no global agreement for the definition of leisure-time physical inactivity [1, 2, 5, 8, 10]. A variety of different kinds of self-administered questionnaires [11, 17, 21, 22, 27] and interview methods [16, 19] have been used in measuring the frequency, duration and intensity of LTPA. Moreover, education systems differ in different countries, and the categorization of educational achievements has also differed between studies. This complicates comparisons and may result in mixed findings between studies. It is also notable that we did not find any longitudinal studies using an objective assessment of LTPA with several time points in analysing the effect of education on LTPA. Hence, further research is needed on the pathway between education and physical inactivity.

Even though LTPA has been reported to be moderately stable over adulthood [25, 26], a decreasing trend of physical inactivity was noticed in this cohort from 1975 to 2011. Our results are in line with several population-based long-term trend reports from the Nordic countries [49–51] and North America [19, 52, 53]. The changes in physical activity over time can be due to issues such as ageing, period effects or changes in the social context due to work and family. Our participants were young adults at the beginning of the study and were nearing retirement or retired at the end of the follow-up. In terms of biological ageing, the ability to exercise does not substantially change across the age range considered here. Diseases may hinder physical activity but may also act as a motive to exercise to improve symptoms and/or to slow the disease’s further progression to disability and premature death [2, 9]. In terms of period effects, we cannot rule out the impact of health promotion campaigns and more access to preventive health care, particularly through occupational health services. Finnish health and social services have become more comprehensive since the 1970s, but teasing out the contribution of individual components is challenging and not possible in our analysis. Social context is probably a greater determinant of physical activity patterns. It is possible that the participants have reported being more active in their leisure time over the years because their leisure time has increased due to changes in their career and work conditions or in their family life over their adult working life. The overall development of LTPA and the association between education and long-term LTPA may also be related to personal life events such as changes in employment status, changes in marital or co-habiting relationships, pregnancy or the birth of a child [19, 54], changes in the social and physical environment such as living areas and transportation systems [55], as well as social desirability in reporting LTPA [42]. Higher educated individuals might have better resources, cognitively and financially, to incorporate physical activity into their daily lives.

In this study, we cannot rule out the effects of the potential gain of further formal or informal education later than 1981 on our results. The effect of later education on the results is supported by earlier findings in which an increase in education level has been associated with an increase in the LTPA level [17] or with the prevention of a LTPA level decrease [19]. However, in our study, the longitudinal association between education and leisure-time physical inactivity remained at the same magnitude as in the cross-sectional analyses. Thus, further education in adulthood would potentially strengthen the observed protective association of higher formal education attainment on the development of long-term physical inactivity. We also cannot rule out some degree of reverse causation if those who are more physically active are more likely to seek further formal or informal training and education.

Many potential pathways may occur between education and healthy behaviours (in this study, the LTPA level). Education may influence work and economic conditions, enhance social and psychosocial resources, and enable a healthy lifestyle and healthy behaviours [56–58], with a potentially stronger effect during bad economic times [57], but with a link to general cognitive ability or intelligence [58, 59]. More educated people have been speculated to read more and internalize health information more efficiently compared to less educated people [58]. Furthermore, higher socioeconomic status, which is most likely associated with higher education, may provide more frequent or more significant opportunities to influence lifestyle [14]. Another possibility is that higher education provides more opportunities to control working schedules to fit LTPA. All of these would be important for the prevention of the epidemic of physical inactivity and the related consequences. Hence, tailored public health campaigns to promote physical activity should be targeted to those with lower education already in early adulthood. Alternatively, the prevention of physical inactivity could be tailored to those with lower education at workplaces or by society through a variety of means.

A unique aspect of this large dataset was the twin study design, which permits controlling for familial factors in the analyses. Earlier studies using discordant MZ pairs have tested hypotheses relating to the causal effects between education and health-related behaviours such as physical activity (i.e. exercise) and reported mainly null associations [15, 36–38]. However, all the previous studies with a co-twin control design have defined or categorized physical activity levels in different ways, preventing any summation regarding the causal effect of education on LTPA. Furthermore, the impact of heritability varies in leisure-time exercise behaviour and leisure-time physical inactivity behaviour [30]. In this study, in the comparison between the associations in the whole cohort and in twin pairs discordant for their LTPA, the protective effect of higher education was similar. However, the point estimates for discordant pairs were very close to those of the analyses of all individuals, suggesting an absence of confounding by familial factors and supporting a direct effect of education on physical inactivity [33, 60]. However, no significant association was detected in the estimates of the MZ twins in our cross-sectional or longitudinal analyses, except in the multivariate analyses in 1990, which is in line with the previous findings on MZ twins [15, 36–38]. Despite this, in this study the within-pair analyses of MZ and DZ twins separately may have lacked the statistical power to detect significant differences. In any case, we cannot completely rule out the impact of familial confounding on long-term inactivity, although the effect of education on physical inactivity was seen throughout the analyses.

This study also has other strengths. Repeating the comprehensive surveys four times provided a unique opportunity not only to follow the same individuals over 35 years but to investigate the long-term stability of and changes in LTPA. The Finnish Twin Cohort is representative of the Finnish adult population [40], but also provides a powerful tool to extend the epidemiological case-control setting into the co-twin design to control for familial confounding. Beyond the familial influences, several covariates with a known influence on LTPA and education were also controlled. The measure of LTPA was based on self-reported data, without any information about domestic (everyday) activities, which may pose a weakness for this study. However, objective measures of LTPA were not available for large surveys in the early 1970s or 80s.

## Conclusions

The proportion of leisure-time physical inactivity decreased in Finnish adults over the 35 years. Each additional year of higher education protects from the development of long-term leisure-time physical inactivity independently of various covariates, but also of familial confounding. The independency from many influential factors and in particular from familial ones suggests that special attention should be targeted those with a lower educational level for promoting physical activity and health throughout adulthood. Furthermore, campaigns and instructions for physical activity promotion should be targeted to those stakeholders and institutions in contact with adolescents and adults with lower education.

## Abbreviations

95 % CI, 95 % confidence intervals; A, active; BMI, body mass index; DZ, dizygotic; I, inactive; ICC, intraclass correlation; LTPA, leisure-time physical activity; MET, metabolic equivalent; MZ, monozygotic; OR, odds ratios
